# Burden of undiagnosed hypertension and associated factors among adult populations in Wolaita Sodo Town, Wolaita Zone, Southern Ethiopia

**DOI:** 10.1186/s12872-022-02733-3

**Published:** 2022-06-27

**Authors:** Tadele Lankrew Ayalew, Belete Gelaw Wale, Bitew Tefera Zewudie

**Affiliations:** 1 Department of Nursing, College of Medicine and Health Science, Wolaita Sodo University, Wolaita, Ethiopia; 2 Department of Nursing, College of Medicine and Health Science, Wolkite University, Wolkite, Ethiopia

**Keywords:** **A**dults, Burden, Ethiopia, Hypertension, Southern Ethiopia, Wolaita Sodo Town

## Abstract

**Background:**

Hypertension is defined as two or more measurements of systolic blood pressure equal to or greater than 130 mm Hg or diastolic blood pressure equal to or greater than 80 mm Hg. At the community level, symptoms of hypertension are not often detected in the early stages and it leads to many people being left undiagnosed with the disease. Undiagnosed hypertension increases the risk of complications like heart failure, kidney failure, myocardial infarction, stroke, and premature death. There is a paucity of studies concerning the burden of undiagnosed hypertension in Ethiopia including the study area. Therefore, this study aimed to assess the burden of undiagnosed hypertension among adults in Wolaita Sodo Town, Wolaita Zone, Southern Ethiopia,2021.

**Methods and materials:**

A community-based cross-sectional study involving 662 study participants was conducted at Wolaita Sodo Town from May 3 to July 3, 2021. A systematic random sampling technique was used to select the total number of participants. The data was entered using Epidata version 3, and analyzed by SPSS version 25 respectively. Binary logistic regression was used to check for a possible association. *P*-values < 0.05 and 95% CI were used on multi-variable analysis as the threshold for the significant statistical association.

**Results:**

A total of 644 have participated in the study giving a response rate of 97.3%. The mean (± SD) age of the study participants was 39.18 (± 10.64) years. This finding showed that the burden of undiagnosed hypertension was 28.8% (95% CI: 24.7–33.2%). Body mass index with overweight (AOR = 2.83, 95% CI: 1.17–6.86), the presence of unrecognized diabetic mellitus (AOR = 1.31 95% CI: 1.11–2.15) habit of alcohol drinking (AOR = 2.91, 95% CI: 1.31–4.48), triglyceride (AOR = 3.48 95% CI: 1.22–9.95), age 31–43 years (AOR = 1.50, 95% CI: 1.02–2.01) were significantly associated factors with undiagnosed hypertension.

**Conclusions:**

The burden of undiagnosed hypertension findings was high. Body mass index with overweight, unrecognized diabetic mellitus the habit of alcohol drinking, triglyceride, and age 31–43 years were the factors with undiagnosed hypertension. These findings suggested that preventing risk factors and screening for hypertension at the community level should be encouraged for early detection, and monitoring of the burden of hypertension with ages more than 30 years old, high body mass index, and undiagnosed diabetic mellitus in the population.

**Supplementary Information:**

The online version contains supplementary material available at 10.1186/s12872-022-02733-3.

## Background

Hypertension is defined as two or more readings of systolic blood pressure measurement of 130 mm Hg or higher or diastolic blood pressure measurement of 80 mm Hg or higher. Around one billion people are affected by hypertension, and it will increase to 1.5 billion by 2025 globally [1]. Undiagnosed hypertension is defined as individuals who were hypertensive but did not report having been told by a health professional that they have hypertension [2]. It is a problem both in developed and developing countries [3, 4]. Among developed countries, around 11 million in the United States of America (USA) [5], Nepales 56.9% [6], in Mexicans 40% [7] in China around 28.8% [8] do not know that they have it and are not being treated. On the other hand, in developing countries, out of 30% of those with hypertension, only 7% were aware and controlled of their hypertensive status before the surveys [9]. It contributes to the rising burden of cardiovascular disease up to 27% [10].

The burden of undiagnosed hypertension among adults in Urban Communities of Southwest Ethiopia was 21.2% [2], in Hawassa 12.3% [11], in Gurage Zone gunchire district, 15.3% [1], in Hosanna town 17.2% [12], in Bahir Dar City among bank workers 24.8% [3]. The existence of a high prevalence of undiagnosed hypertension definitely leads to a substantial-high risk for morbidity and mortality from potentially preventable complications of hypertension such as stroke renal failure, myocardial infarction, heart failure, and premature death from heart disease and the economic burden on the population in the world [5, 12]. Globally, if the current undiagnosed hypertension levels persist and are untreated, it will nearly cost more than US$1trilion, and indirect costs could be as high as US$3.6 trillion annually [4, 13].

Different factors contribute to undiagnosed hypertension status. Those are smoking, unhealthy diets such as high intake of fat, salt, and refined sugars stress, physical regular inactivity, BMI > 25, older age, being alcohol drunker, and increased total cholesterol were significantly associated factors with undiagnosed hypertension [10, 14].

Early diagnosis and treatment are essential for hypertension management, however, in developing countries like Ethiopia, the majority of the communities are not aware of their hypertension status and are left undiagnosed, untreated, and uncontrolled [14]. A systematic review and meta-analysis done in sub-Saharan Africa among the general population showed that only 27% of individuals know their hypertension status [15]. This indicates that a large population in developing countries with hypertension is left undiagnosed and untreated [10]. In Ethiopia, the design/plan of HTN intervention considers only the diagnosed/known hypertensive individuals but ignores the larger population of un-diagnosed hypertensive. This has potentially severe consequences in the country, as a large proportion of those with hypertension probably remain undiagnosed, untreated, or inadequately treated.

Therefore, to halt the complication of hypertension, surveillance of undiagnosed hypertension is important. Although there were few studies, there is no adequate scientific evidence on the burden as well as on the correlates of undiagnosed hypertension in Ethiopia, particularly in the study area. Therefore, this study aimed to assess the burden of undiagnosed hypertension and associated factors among adult populations in Wolaita Sodo town, South Ethiopia.

## Methods and materials

### Study area and study period

This study was conducted in Wolaita Sodo town from May 3 to July 3, 2021. Wolaita Sodo town is found in Wolaita Zone in South Nations Nationalities people of Region (SNNPR) of Ethiopia; which is located 346 km far from Addis Ababa, the capital city of Ethiopia. It has 3 sub-cities and 17 administrative units (Kebele). Based on the 2020 population projection conducted by central statistical agency of Ethiopia (CSA), the town has a total population of 534,597, and 110 454 households which results in an average 4.48 persons to a household. There are 3 hospitals, 3 health centers,11 health posts and more than 21 private health institutions providing health services in the town.

### Study design and population

A community-based cross-sectional study was conducted on the are adult populations aged ≥ 18 years at Wolaita Sodo town. All adult residents who had lived at least six months in the town were encircling and used this study. On the other hand, those adults previously diagnosed with hypertension hospitalized patient, pregnant mothers, physical deformities person (kyphosis, Sclerosis), a mentally ill person was excluded from the study to control for the occurrence of misclassification of physical measurements like BMI (wt./ht2), waist circumstance, and waist to hip ratio due to Kyphotic posture, and pathological hardening of tissue by kyphosis, and Sclerosis respectively.

### Sample size determination and sampling procedures

We computed the sample size of this study by using a single population proportion formula including assumptions of z^a/2^ = 1.96 at 95% C level, the margin of error_=_4%, p (population proportion) with a prevalence of undiagnosed hypertension was 21.2% studied in southwest Ethiopia [14], and adding 10% non-response rate and multiplied by 1.5 design effect the final sample size = 662.

**Fig. 1:**
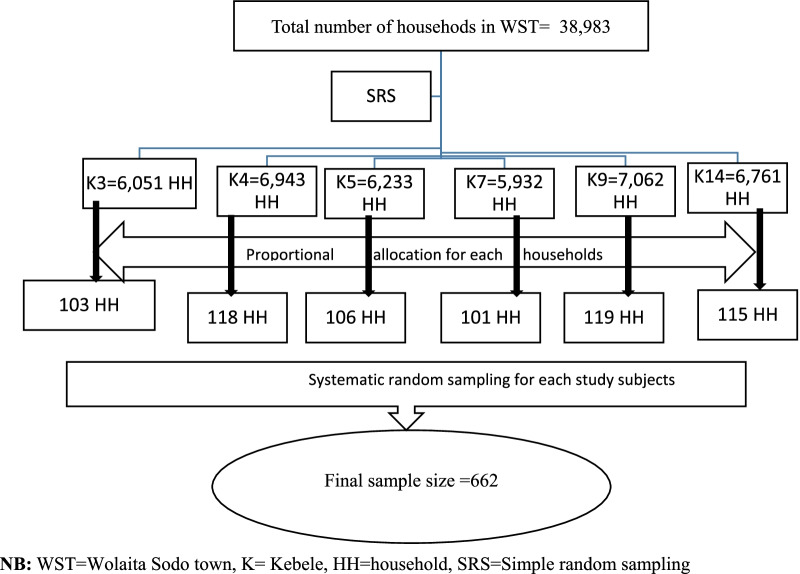
Sampling procedure of undiagnosed hypertension and its related variables in adult populations in Wolaita Sodo, South Ethiopia, 2021

A multistage sampling technique was employed to recruit the study participants. In the first stage, using a simple random sampling technique, we selected six kebeles (the lowest level of administration in Ethiopia) out of 17 kebeles found in the town. In the second stage, households were selected from the kebeles by using a systematic random sampling technique from the family folder found at the health posts (the government providing community health services in Ethiopia). Then, we randomly selected one adult aged ≥ 18 years old from each household by using the lottery method (Fig. 1).

### Data collection procedures and measurement

The WHO STEPS Questionnaire [16] was adapted based on the study objective and the local context of the study setting to collect the data. The data collection tool was initially prepared in English, then translated to Amharic and then Wolayitegna. A questionnaire includes socio-demographic behavioral, physical, and biochemical measurements of the study participants.

### Blood pressure measurement

Blood pressure was measured while the study participant was in a sitting position by using an Aneroid Sphygmomanometer after five minutes of rest. They were barely supported and measured BP with appropriate cuff size for the patient arm. Subsequent measurements were done five minutes later from the first measurement. The average of two separate BP measurements was taken in the final analysis. blood pressure having a systolic blood pressure (SBP) ≥ 130 mmHg or diastolic blood pressure (DPB) ≥ 80 mm Hg on at least two occasions [17].

### Physical measurement

The waist circumference(WC) was measured at the midway between the lowest costal margin at the mid-clavicular line and the anterior superior iliac spine using fixed tension tape. WC values > 80 cm for women and > 94 cm for men were considered high according to WHO recommendations [18]. The height and weight were measured to calculate body mass index. Using a stadiometer (Seca, Germany) the height of the study participant was measured to the nearest 0.1 cm. with the Frankfurt plan position and the four points (heel, calf, buttock, and shoulder) touching the vertical stand and their shoes taken off with a checked stadiometer. Weight was measured with light clothes and shoes by using a checked digital weight scale to nearest to 0.1 kg. BMI was calculated by dividing weight in kilograms of adult height in meters squared formula. BMI < 18.5 kg/m^2^ was considered as underweight, 18.5–24.9 kg/m^2^ was normal, 25–29.9 kg/m^2^ was overweight, and ≥ 30 kg/m^2^ was obese [19].

### Biomedical measurements

Low-density lipoprotein (LDL), high-density lipoprotein (HDL), random and fasting blood glucose, triglyceride (TG) level measurements were done. Five milliliters of venous blood were collected from adults to determine their fasting blood glucose levels. Serum was carried out in ABX Pentra 400 Automated Chemistry Machine at wolaita Sodo comprehensive specialized hospital chemistry laboratory to determine lipid profile, and blood glucose level. The low–density lipoprotein level of the study participants was calculated by using the Friedewald formula. The optimal level of LDL was less than 100 mg/dl and greater than 100 mg/dl was defined as high. HDL is considered as desirable if it is > 40 mg/dl for men and > 50 mg/dl for women [20].

Diabetic mellitus was diagnosed based on the ADA and IDF classification criteria with random blood glucose (RBG ≥ 200 mg/dl) and fasting blood glucose (FBG ≥ 126 mg/dl) [21]. Triglycerides level was considered as normal when it was less than 150 mg/dl and if greater than 150 mg/dl considered high [22]. All laboratory values were confirmed by a laboratory technologist in wolaita sodo comprehensive specialized hospital.

### Study variables

The dependent variable considered in this study was the burden of undiagnosed hypertension. Independent variables measured in this study were sociodemographic characteristics such as age, sex, education, occupation, family size, clinical-related factors like anthropometric measurements, waist to hip ratio, waist circumference, blood pressure, and BMI, and Biochemical tests like triglyceride and blood glucose, and lifestyle-related factors which include smoking, alcohol drinking, chat chewing physical activity.

### Operational definition

- *Hypertension*: Is a high blood pressure having a systolic blood pressure (SBP) ≥ 130 mmHg or diastolic blood pressure (DPB) ≥ 80 mm Hg on at least two occasions [3].
- *Undiagnosed hypertension*: Refers to individuals who were hypertensive but did not report having been told by a health professional that they have hypertension [1].
- *Comorbid disease*: a chronic disease with a confirmed diagnosis of a disease other than hypertension [23].
- *Regular physical exercise*: Performing physical activity three days/week for 20–30 min duration [24].
- *Alcohol intake*: individuals who take more than three units of alcoholic beverage per day [25].
- *Smoking*: individuals who has a history of smoking or current smokers [25].
- *Regular khat chewing*: which is defined as khat chewing at least once per weekly for the past one year or more by adopting the definition used for other substances [26].

### Data quality control

Six trained professional nurses were selected based on qualification, prior experience, and ability to speak the local language. Training on interviewing approach, sample talking, anthropometric measurement, and data recording were given to the data collectors for 2 days before the real data collection. We have done a pretest on 5% of the total sample size to check the reliability of the tool in Hossana town 2 weeks before applying it to the final data collection. Close supervision by the investigators was done during the interview and measurements by data collectors. Daily basis reviews and checkups were done on each questionnaire for completeness, accuracy, and consistency of the collected data before data entry.

### Data analysis

Data entry and analysis were performed using SPSS version 25 statistical software. Descriptive statistic were computed to present frequency distributions. Bivariable logistic regression analysis was done to identify candidate variables for multivariable logistic regression analysis. All variables with a *p*-value ≤ 0.25 in the bivariable logistic regression analysis were included in the multivariable model. A multivariable logistic regression model was used to identify the independent correlates of the burden of undiagnosed hypertension. Odds ratio (ORs) with 95% confidence intervals were calculated. The result was declared statistically significant if the p-value was below 0.05 and the 95% 95% CI did not in line with the null value. The Hosmer-Lemeshow goodness of fit assumptions was used to assess to check the application of multiple logistic regression.

## Results

### Socio-demographic, behavioral and clinical-related characteristics of respondents

Of a total of 662 study participants, 644 have participated in the study giving a response rate of 97.3%. Nearly half of 330 (49.9%) were age category of 30–49 years with a mean (± SD) age of the study participants was 39.2 (± 10.6) years. More than two-thirds of participants' family size was greater than three (Table 1).

**Table 1:** Sociodemographic, Behavioral, Anthropometric, and Biochemical characteristics of study participants in Wolaita Sodo town, Wolaita Zone, South Ethiopia, in 2021 (N = 644)

Variable	Frequency	Percent (%)	Variable	Frequency	Percent (%)
**Sex**			**Family size**		
Male	247	38.4	< 3	181	28.1
Female	397	61.6	> 3	463	71.9
**Age groups (years)**			**Marital status**		
18–30	287	44.6	Married	487	75.6
31–43	240	37.3	Single	40	6.2
44–56	69	10.7	Divorced	101	15.7
> 57	48	7.5	Windowed	16	2.5
**Occupation**			**Educational status**		
Unemployed	69	10.7	Primary school	48	7.5
Employed	238	36.9	Secondary school	238	36.9
Merchants	289	44.9	Illiterate	69	10.7
Farmer	48	7.5	Diploma and above	289	44.9
**Smoking**			**Annual income (ETB)**		
Yes	85	13.2	< 2000	289	44.9
No	501	77.8	2001–4000	238	37
**Alcohol use**					
Yes	506	77.8	> 4001	69	10.7
			**Regular exercise**		
No	80	13.2	Yes	463	71.9
**Khat chewing**					
Yes	501	23.8	No	181	28.1
			**Duration of regular exercise per day**		
No	85	67.2	< 30 min	181	28.1
**Unrecognized DM**					
No	181	18.1	≥30 min	463	71.9
			**LDL**		
Yes	463	81.9	Normal	363	56.4
**BMI**					
Normal	289	44.8	High	281	43.6
			**Triglyceride**		
Underweight	238	36.9	Normal	501	77.8
Overweight	69	10.7	High	85	13.2
			**Waist to hip**		
Obese	48	7.5	Normal	384	59.6
			Overweight	132	20.5
			**HDL**		
			Desirable ≥ 40	463	71.9
			Low < 40	181	28.1

### The burden of undiagnosed hypertension and factors associated with it

In the current study,the burden of undiagnosed hypertension was 185 (28.8% = 95% CI: 24.7%, 33.2%). Variables such as marital status, alcohol drinking, sex, age, chat chewing, smoking, and having diabetes mellitus, were significantly associated with undiagnosed hypertension in bivariable logistic analysis at *p*-value < 0.25. In multivariable logistic analysis only six variables, unrecognized diabetes mellitus, alcohol drinkers, BMI with overweight, triglyceride, and age were significantly associated with the burden of undiagnosed hypertension at a *p*-value < 0.05.

Study participants who had unrecognized diabetes mellitus were 1.31 (AOR = 1.31 95% CI: 1.11–2.15) times more likely to have hypertension than their counterparts. In this study, the odds of having HTN were tripled by 2.91 (AOR = 2.91, 95% CI: 1.31–4.48) among alcohol drinkers than nondrinkers. BMI with overweight 2.83 (1.17–6.86), triglyceride 3.48 (AOR = 3.48 95% CI: 1.22–9.95). Finally, participants whose age was 31–43 years were 1.50 (AOR = 1.50, 95%CI: 1.02–2.01) times more likely to have hypertension than other age groups (Table 2).

**Table 2:** Multi-variable logistic regression analysis model of factors associated with undiagnosed hypertension among adults in Wolaita Sodo town, south Ethiopia, 2021

Variable	Category	Undiagnosed HTN		COR	AOR
		Yes	No	(95% CI)	(95% CI)
Sex	Male	145 (22.51%)	102 (77.47%)	1	1
	Female	227 (35.24%)	170 (64.75%)	0.31 (0.16–0.63)	0.44 (0.18–1.07)
Age	18–30	115 (64.3%)	172 (47.9%)	1	1
	**31–43**	**182 (28.26%)**	**58 (9.1%)**	**1.89 (1.30–2.75)**	1.50 (1.02–2.01)*
	44–56	40 (6.3%)	29 (4.50%)	2.48 (1.11–5.56)	1.15 (0.39–3.33)
Unrecognized DM	> 57	29 (4.50%)	19 (2.95%)	2.26 (1.57–3.27)	1.31 (0.79–2.15)
	No	80 (12.42%)	101 (15.68%)	1	1
	**Yes**	**263 (40.83%)**	**200 (31.05%)**	**2.26 (1.57–3.27)**	1.31 (1.11–2.15)*
BMI	Normal	117 (64.3%)	172 (47.9%)	1	1
	Under weight	**180 (28.26%)**	**58 (9.1%)**	2.26 (0.91–5.66)	2.48 (0.89–6.97)
	Overweight	40 (6.3%)	29 (4.50%)	**2.99 (1.38–6.49)**	2.83 (1.17–6.86)*
	Obese	30 (4.50%)	18 (2.95%)	0.31 (0.16–0.63)	0.44 (0.18–1.07)
Alcohol drink	No	145 (22.51%)	102 (77.47%)	1	1
	**Yes**	**227 (35.24%)**	**170 (64.75%)**	**3.12 (2.06–6.74)**	2.91 (1.31–4.48)*
Cigarette smoking	No	145 (22.51%)	102 (77.47%)	1	1
	Yes	227 (35.24%)	170 (64.75%)	3.06 (1.57–5.95)	0.95 (0.39–2.27)
Khat chewing	No	145 (22.51%)	102 (77.47%)	1	1
	Yes	227 (35.24%)	170 (64.75%)	3.04 (1.94–4.78)	2.33 (0.80–4.51)
LDL	Normal	80 (12.42%)	101 (15.68%)	1	1
	High	263 (40.83%)	200 (31.05%)	3.16 (3.16–1.65)	2.21 (0.82–5.95)
Triglyceride	Normal	169 (91.4%)	332 (82.8%)	1	**1**
	**High**	**16 (8.6%)**	**69 (17.2%)**	**2.20 (2.20–1.24)**	3.48 (1.22–9.95)*
Waist to Hip	Normal	149 (80.5%)	235 (58.6%)	1	1
	Over weight	18 (9.7%)	114 (28.4%)	4.016 (2.34–6.87)	2.87 (0.14–7.18)
HDL	Desirable ≥ 40	80 (12.42%)	101 (15.68%)	1	1
	Low < 40	263 (40.83%)	200 (31.05%)	0.31 (0.16–0.63)	0.44 (0.18–1.07)

Bold indicates significantly associated variables between independent and outcome variables

Significant at *P ≤ 0.001, 1 = constant, CI = Confidence Interval, COR = Crude odds Ratio, AOR = Adjusted Odds Ratio, BMI = body mass index, HTN = hypertension, LDL = low density lipoprotein

## Discussion

This study aimed to determine the burden and associated factors of undiagnosed hypertension among adults in Wolaita Sodo town, Southern Ethiopia We found that undiagnosed hypertension among adults in this study area was 28.8% (95% CI: 24.7–33.2%). This finding was higher than a study conducted among adults in urban communities of southeast Ethiopia [14], studies done in are Ireland [27], and studies done in Hawassa [11], Bahardar [3], in the Gurage zone, Gunchire district [7], in Hosanna town [8]. The possible justification might be due to differences in socio-demographic characteristics of study participants. Our study was conducted on the general adult population, whereas most other studies were done on populations with a greater risk for hypertension than the general population.

This study was consistent with a study conducted among the Middle-Aged and Elderly Chinese Population [8], among Secondary School Teachers in Bahir Dar City Administration, Northwest Ethiopia [28], in urban areas of Uganda [29].

However, this study’s finding is much lower than studies conducted in a systemic review study done in Ethiopia [13], eastern Tanzania, Cameroon, Malaysia [30], and a study conducted among adults in the Nepalese population [6]. The above discrepancy might be the difference in inclusion criteria (the above-mentioned studies used all previously diagnosed as hypertension and undiagnosed individuals but this study assesses only undiagnosed/ unknown hypertension only. The other possible justification could be a difference in the study setting, and different lifestyles as well as sociodemographic characteristics of respondents. The variation might be due to hypertension cut point, duration, and the difference in socioeconomic and ethnic factors.

Study participants who had unrecognized diabetic mellitus were 1.31 times more likely to have hypertension than their counterparts. In this study, the odds of having HTN were tripled among alcohol drinkers than nondrinkers. Body mass index with overweight three times more likely to have hypertension. Study participants who have high triglycerides were three times more likely to have hypertension other than normal triglycerides. Finally, participants whose age was 31–43 years were 1.50 times more likely to have hypertension than other age groups.

This study showed that adult populations who had unrecognized diabetic mellitus were significantly associated with undiagnosed hypertension. This finding is consistent with a study done in eastern Ethiopia, Harar jugal hospital which revealed that increased glucose levels in the blood were significantly associated with the magnitude of hypertension and northeast Ethiopia [2, 3]. This finding is also supported by a previous study done on the burden of hypertension among adult persons which demonstrated that diabetic mellitus was a strong correlates of hypertension [12]. The other justification could be the shared risk factors like obesity and physical inactivity may contribute to hypertension risks.

In this study, age between 31 and 43 years old was independently associated with hypertension. This finding is consistent with studies done in the northeast, and southern Ethiopia [2], Malaysia [31], the United States, Uganda [32], and a cross-sectional study in Senegal [33]. A plausible explanation could be that with advancing age hypertension is more likely diagnosed than in younger persons who may have less contact with the medical system.

Finally, this study finding showed that participants who had alcohol drinking experience were significantly associated with hypertension. This finding agrees with studies done in Kenya [32], and Eastern Ethiopia, Jugal hospital [1, 11]. This finding is also supported by clinical studies which stated that chronic alcohol consumption of more than three drinks per day contributes to the incidence of hypertension [8]. This could be due to chronic consumption of alcohol more than 20 g of ethanol per day leads to dose-dependent increased blood pressure and chronic hypertensive effect manifested due to shifting of calcium into vascular smooth muscle cells which coupled with an outward shift of magnesium [1]. In Ethiopia, the design/plan of HTN intervention considers only the diagnosed/known hypertensive individuals but ignores the larger population of un-diagnosed hypertensive. This has potentially severe consequences in the country, as a large proportion of those with hypertension probably remain undiagnosed, untreated, or inadequately treated. Therefore, this findings suggested that preventing risk factors and screening for hypertension at the community level should be encouraged for early detection, prevention, and monitoring of the burden of hypertension. The prevention of risk factors and routine screening techniques should help to promote early detection, prevention, and treatment of hypertension. During routine adult service providing, health facilities should consider assessing hypertension and possible risk factors.

## Conclusion

The burden of undiagnosed hypertension findings was high. Body mass index with overweight, unrecognized diabetes milletus, the habit of alcohol drinking, triglyceride, and age 31–43 years were the factors with undiagnosed hypertension. This findings suggested that preventing risk factors and screening for hypertension at the community level should be encouraged for early detection, prevention, and monitoring of the burden of hypertension with aged more than 30 years old, high body mass index and undiagnosed diabetic mellitus in the population..

### Limitation of the study

The community based nature and large sample size used for this study was its strengthen. However, the study did not consider acute and chronic illness other than diabetic mellitus and hypertension.Since the study design is cross-sectional, the cause and effect relationship cannot be established. Moreover,factors like diatary factors, wealth index, DALYS, cholesterol level, and as well as genetic factors have not been assessed in this study. Data about lipid present and renal function can not be obtained. This study has a flaw in that it uses an aneroid sphygmomanometer for blood pressure measurement, which is not a validated blood pressure measurement instrument.

## Supplementary Information

**Additional file 1**. This manuscript includes all important data.

## Data Availability

The data set used and or analyzed during the current study are available from the corresponding author on reasonable request.

## Abbreviations

DM: Diabetes Mellitus
HTN: Hypertension
WHO: World Health Organization
