# Supplementary material for: Burden of undiagnosed hypertension and associated factors among adult populations in Wolaita Sodo Town, Wolaita Zone, Southern Ethiopia
Source: BMC Cardiovasc Disord. 2022 Jun 27;22:293. doi: 10.1186/s12872-022-02733-3 (PMC9238150; doi:10.1186/s12872-022-02733-3)
Supplement: Supplementary file 1 — Additional file 1. This manuscript includes all important data. [file 12872_2022_2733_MOESM1_ESM.docx]

**Sample size determination and sampling procedures**

We computed the sample size of this study by using a single population proportion formula including assumptions of z^a/2^=1.96 at 95% C level, the margin of error_=_4%, p (population proportion) with prevalence of undiagnosed hypertension was 21.2% studied in southwest Ethiopia(10), and adding 10 % non-response rate and 1.5 design effect. $n=\frac{z_{\frac{a}{2}}^{2} (p(q)}{d^{2}}$= $\frac{{(1.96)}^{2}(0.212(1-0.212)}{({0.04)}^{2}}$ = 401, and adding 10% non-response rate and multiplied by 1.5 design effect, final sample size =662.
